# Control strategies of 3-cell Central Pattern Generator via global stimuli

**DOI:** 10.1038/srep23622

**Published:** 2016-03-29

**Authors:** Álvaro Lozano, Marcos Rodríguez, Roberto Barrio

**Affiliations:** 1 Centro Universitario de la Defensa de Zaragoza, Academia General Militar, Ctra. Huesca s/n. E-50090, Zaragoza, Spain; 2 Universidad de Zaragoza, Departamento de Matemática Aplicada, Pedro Cerbuna, 12. E-50009, Zaragoza, Spain

## Abstract

The study of the synchronization patterns of small neuron networks that control several biological processes has become an interesting growing discipline. Some of these synchronization patterns of individual neurons are related to some undesirable neurological diseases, and they are believed to play a crucial role in the emergence of pathological rhythmic brain activity in different diseases, like Parkinson’s disease. We show how, with a suitable combination of short and weak global inhibitory and excitatory stimuli over the whole network, we can switch between different stable bursting patterns in small neuron networks (in our case a 3-neuron network). We develop a systematic study showing and explaining the effects of applying the pulses at different moments. Moreover, we compare the technique on a completely symmetric network and on a slightly perturbed one (a much more realistic situation). The present approach of using global stimuli may allow to avoid undesirable synchronization patterns with nonaggressive stimuli.

The production of coordinated and rhythmic behaviors in organisms, such as chewing, respiration, walking, crawling and swimming, is a fundamental question in the study of motor control and neuroscience. Many of these behaviors are driven by central pattern generators (CPGs), which are groups of neurons (small biological neuron networks) whose interactions can produce rhythmic patterns^1^,^2^,^3^,^4^,^5^ (like in locomotive patterns^6^,^7^,^8^ or in the direct-reverse flow of the circulatory system in leeches^9^,^10^) even in isolation from motor and sensory feedback from limbs and other muscle targets.

Although anatomical details of CPGs are only known in a few cases, they have been shown to originate from the spinal cords of various vertebrates and to depend on relatively small and autonomous neuron networks. The classical view of CPGs, as specific networks of neurons dedicated to this function alone, has been supported by numerous data mostly obtained from central nervous systems of invertebrates. In these cases, it is possible to identify many of the key neuronal elements composing a pattern generator, leading to an easier analysis. Besides, it is possible to record and to biophysically analyze these neurons and their synaptic interactions. For instance, swimming in the medicinal leech, *Hirudo medicinalis*, is driven by a CPG composed of a set of eight pairs of cells and one unpaired cell per ganglion^11^,^12^. The CPG for heartbeat in leeches consists of seven identified pairs of segmental heart interneurons and one unidentified pair^13^.

A key point in live organisms is that they must adapt their behavior to meet the needs of their internal and external environments. Individuals vary in their responses to stroke and trauma, hindering predictions of results. An explanation might be that neuron circuits contain hidden variability that becomes relevant only when those individuals are challenged by injury^14^. CPGs, as part of the neuronal circuitry of an organism, can be modulated or controlled (neuromodulation) to adapt to the environment and to the organism’s needs.

Mathematical modeling is essential to analyze CPGs and, although the real circuitry involved in a particular CPG is far from being known, these models generate meaningful hypotheses about the network function. A deep study of simplified models arises as a natural first step in one of the main challenges of the new century—comprehending brain activity. Unraveling the mechanisms of such an incredibly complex conglomerate requires to fully understand the dynamics of its basic elements—neurons and small neuron circuits or motifs. Such motifs share same characteristics detected in oscillator networks^15^,^16^,^17^. Therefore, mathematical studies of reduced CPG models produce useful insights, shedding light onto some operational principles of biological CPG networks.

The study of synchronization patterns in CPGs has become an interesting growing discipline since it provides details of the different tasks a CPG may control. We remark that several of these synchronization patterns of individual neurons are related to some undesirable neurological diseases, and it is believed to play a crucial role in the emergence of pathological rhythmic brain activity in different diseases, like Parkinson’s disease, essential tremor, and epilepsy^18^. The theoretical study of the synchronization patterns associated to these diseases, and how to control them, is a mayor goal in neuroscience. For instance, the development of techniques for suppression of the undesired neural synchrony constitutes an important clinical problem. Technically, this problem can be solved by implanting microelectrodes into the impaired part of the brain with subsequent electric stimulation^18^. A recently studied technique is Deep Brain Stimulation (DBS)^19^. The DBS technique, based on a global stimulation of the neuronal circuit, has the main objective of reestablishing desynchronization of the network (or another synchronization pattern) via a pulse train, whose parameters are selected by the neurosurgeon to decrease the disease symptoms. The results of that study confirm what is expected from the Gate Control Theory^20^, the synchronization of neuronal activity obstructs information flow in brain structures, whereas, the desynchronization allows the flow. Therefore, the development and study of mathematical models is crucial, and simulation of models of CPGs could provide new treatments and therapies.

In mathematical control of ordinary differential systems several approaches have been proposed^21^ using different optimization techniques. An active area in dynamical systems is the control of chaotic systems^22^ by means of the stabilization of some particular unstable periodic orbits which foliate the chaotic invariant set (the E. Ott, C. Grebogi and J. A. Yorke approach^23^), or by an appropriate continuous controlling signal injected into the system (the Pyragas method^24^). These approaches are not suitable for biological networks since it is not always possible to change some parameters since they are fixed by the living environment and its is necessary to isolate the Poincaré section to locate unstable periodic orbits and to compute the precise perturbations necessary to attain stability. Another control method consists of varying one parameter (external electrical current, a parameter that really can be tuned) of a single neuron of the model^25^. However, since that parameter corresponds to an external electrical current and the neurons of the CPG are supposed to be extremely close, it seems unrealistic to suppose that the current does not affect the remaining neurons. In our case, we try to find ways to control the CPG using short and weak global pulses. That is, we show how, with a suitable combination of global inhibitory and excitatory stimuli of the complete network, we can switch between different stable bursting patterns in small CPG neuron networks. This approach may open new ways of controlling undesirable synchronicity patterns in CPGs.

## Results

### The Mathematical Neuronal Model

**Figure 1:**
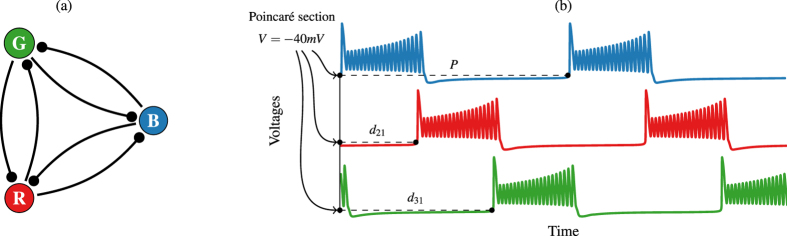
(**a**) Scheme of the 3-cell network with inhibitory synapses. (**b**) Definition of the delays between neurons with blue neuron as the reference burster.

The basic neuron circuit we are going to consider is a model represented by a three node network (Fig. 1a). This model is the main building block that makes up more complex CPG circuits (we note that many anatomically and physiologically diverse CPG circuits involve a three-cell motif, including the spiny lobster pyloric network, the *Tritonia* swim circuit, and the *Lymnaea* respiratory CPGs).

The nodes of the network represent neurons which can present two different states, active (bursting or spiking) or inactive (quiescence). The edges of the network represent the synaptic connections between neurons. The synaptic connection is directed from the axon of a pre-synaptic neuron to the dendrite of a post-synaptic one. Therefore, we consider two different edges between each pair of neurons representing this biological circuit. In our case each node of the network will model a leech interheart neuron^26^,^27^, an inhibitory neuron model derived from the Hodgkin-Huxley formalism^28^:


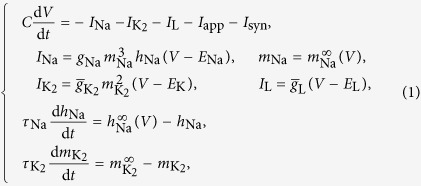


where the dynamics of the gating variables are determined by the experimentally calibrated Boltzmann functions:


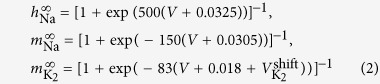


(see ref. 27 and references therein for an exhaustive description of the model and the *Methods* section for the values of the parameters used in our simulations).

**Figure 2:**
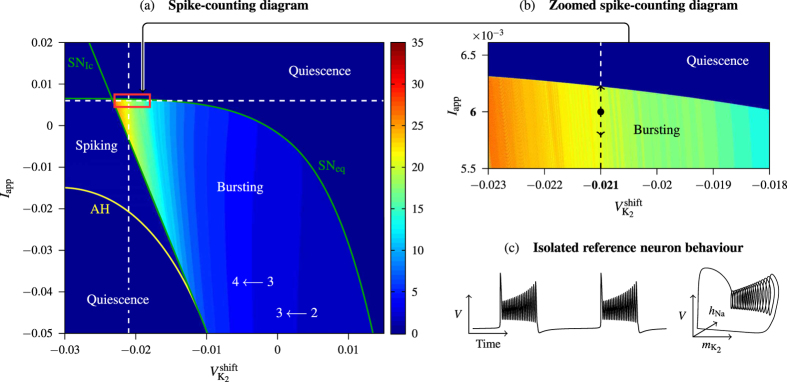
(**a**) 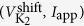-biparametric sweep using the spike-counting method (color-coded bar on the right for spike range) with superimposed bifurcation lines, for Andronov-Hopf and saddle-node bifurcations, demarcating the regions of bursting, tonic-spiking and quiescent activity in individual neurons. (**b**) Magnification of the selected region. Increasing the *I*_app_ current (upwards arrow) inhibits the neuron and vice versa. (**c**) Time series of the voltage variable of the reference burster and a plot of the periodic orbit.

There are two main parameters controlling the activity in the model of each individual burster: the external current *I*_app_ that affects the fast voltage dynamics, and the parameter 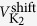, which is the deviation from the experimentally averaged value, *V* = −0.018 *V*, corresponding to the half-activated gating channel for the slow potassium current. Both *I*_app_ and 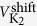 are independent bifurcation parameters. Their variations make the neuron dynamics evolve and switch between tonic spiking, bursting and quiescence. In terms of dynamical system theory, these regimes are associated, respectively, with stable one- and two-time scale periodic orbits (that can become chaotic at bifurcations) and equilibrium states in the phase space of the model. Figure 2a represents the 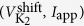-biparametric sweep of the isolated neuron model obtained with the spike-counting method^29^. Its core is the number of spikes between contiguous quiescent periods for the given parameter values. The spike number is encoded according to the colorbar on the right. This method allows us to identify stability windows with constant spike numbers, as well as to detect the borders where the spike number changes. We can see these structures separated by spike-adding bifurcations^30^,^31^ in Fig. 2 with clearly demarcated regions corresponding to bursting, tonic spiking and quiescence states. The sweep diagram is overlaid with several key curves (obtained by the parameter continuation package MatCont^32^) that correspond to bifurcation transitions between different activity states^27^. These are the saddle-node bifurcation of equilibria between hyperpolarized quiescence and bursting (*SN*_*eq*_), saddle-node bifurcation of limit cycles on the tonic-spiking and bursting boundary (*SN*_lc_) and the Andronov-Hopf bifurcation on the boundary between depolarized quiescence and tonic spiking (*AH*). The combined bifurcation diagram serves as a “road map” for individual ingredients (isolated neurons) used to build a suitable model of a multifunctional CPG circuit^25^. Once we have the global picture we magnify a region of bursting behaviour (Fig. 2b), centered at the selected parameter values for our leech neurons: 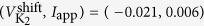. Around these values the neuron is a burster with 21 spikes per period (Fig. 2c). Note that small perturbations of the parameter values move the neuron to a quiescence state or change the number of spikes (the vertical line in Fig. 2b).

The three neurons of the network are reciprocally coupled via the *I*_syn_ term, which models fast and weak chemical synapses using the fast threshold modulation scheme:


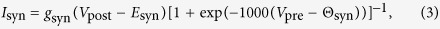


where *V*_post_ and *V*_pre_ are the voltages of the post- and pre-synaptic cells. Following refs 25,26,33, the study of the model can be done by analyzing fixed points of the Poincaré return maps to obtain the phase lags between the bursting periods of the neurons. Taking the blue neuron as the reference burster, we define the phase delays sequence 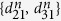 as described in Fig. 1b. We normalize these values dividing them by the period *P* (lapse between bursts) of the blue neuron and we consider the normalized value modulo 1 (phase lags denoted by *φ*_21_ and *φ*_31_ resp.). Thus, we have a discrete dynamical system on a torus^26^ and so, for any initial state of the network, we can compute the corresponding sequence of phase lags on the phase torus, that we represent in a 2D plot.

**Figure 3:**
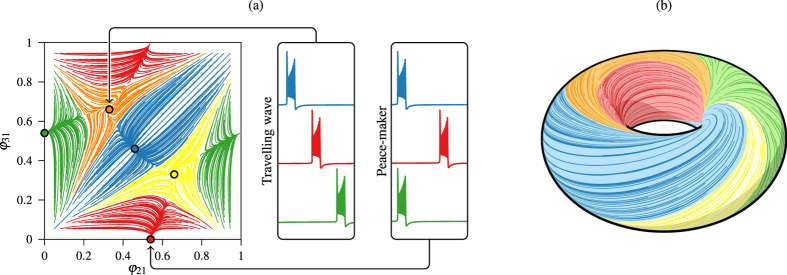
Phase torus. (**a**) Planar (2D) representation of the phase torus where *x*-axis represents the lag of the red neuron with respect to the blue neuron and *y*-axis the lag of the green one with respect to the blue one. Stable fixed points are marked. (**b**) 3D representation of the phase torus.

A stable pattern of the network will produce a constant sequence of lags, that is, a fixed point in the phase torus. In Fig. 3a we have marked 5 stable points, corresponding to the main 5 stable patterns this network can achieve. The points Blue *P*_1_ = (0.45, 0.45), Red *P*_2_ = (0.54, 0) and Green *P*_3_ = (0, 0.54) correspond to the situation where two neurons are fully synchronized. The other neuron, called the *pace-maker*, has a lag of 0.54 with respect to the group of two and gives the color to the point in our representation. The points Yellow *P*_4_ = (0.66, 0.33) and Orange *P*_5_ = (0.33, 0.66) correspond, respectively, to a counter-clockwise and a clockwise *traveling wave* on the network of Fig. 1a where each neuron starts its duty cycle after the previously excited neuron with a lag of 0.33. Near point (0, 0) new stable patterns can appear, where the 3 neurons are bursting almost at the same time^25^. Each line of the plot represents an integration of the model starting from different initial lags. The lines have been colored according to the stable state the network reaches. Figure 3b is a 3-dimensional representation of the phase torus^26^. Note that varying the parameters of the system the network experiments different bifurcations that change the number and type of patterns^34^.

### Control strategies via global stimuli

As previously shown, a CPG exhibits different stable bursting patterns (multistability) which may correspond to biological functions such as locomotive patterns^6^,^7^,^8^ or the direct-reverse flow of the circulatory system in leeches^9^,^10^. Therefore, jumping from one stable pattern to another in order to change the biological response is an intrinsic mechanism of the animal. The natural question that arises is whether we can force those changes by applying external stimuli to the network.

The authors of some recent papers (see ref. 25 and references therein) propose to force those changes by varying the parameter *I*_app_ for a single neuron of the model or, more generally, varying that parameter simultaneously in different neurons with different currents. However, since that parameter corresponds to an external electrical current and the neurons of the CPG are supposed to be extremely close, it seems unrealistic the use of such technique.

We propose the strategy of applying *the same external current* to all neurons of the CPG *during the same time*. More precisely, we propose to apply an inhibitory pulse followed by a excitatory one. Along this paper, the control technique is composed of two opposite pulses, although the cases of two inhibitory or two excitatory pulses are also shown. This approach is more realistic since an external current should affect in a similar way all the neurons of a CPG inside of a living being, as in the Deep Brain Stimulation (DBS)^19^ technique.

**Figure 4:**
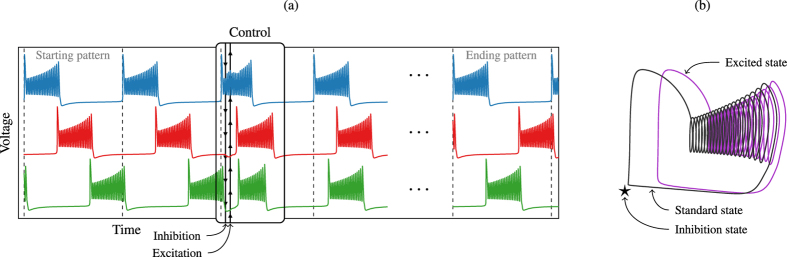
Control technique. (**a**) In the timeseries of voltages, black arrows represent the inhibitory and excitatory pulses applied to the network. (**b**) The modification of the stable periodic solution of the system (each individual neuron) due to the impulses. The standard behaviour of a detached neuron is depicted in black (cf. Fig. 2c). The global attractor (equilibrium point) of the inhibited system is marked with a black star, and the periodic orbit associated to the excited system is drawn in violet.

Figure 4a shows a CPG bursting according to a stable pattern (Orange fixed point *P*_5_ = (0.33, 0.66) in phase space Fig. 3). At a certain instant we apply the first pulse (of length much shorter than the period) and after it the second pulse is applied. We can observe that, after those stimuli, the CPG is no longer bursting according to a stable pattern. Moreover, the number of spikes in the bursting activity is altered giving shorter and/or longer duty cycles. During the first inhibitory pulse, which acts as a temporal change of parameters up in the vertical straight line in Fig. 2b, the attracting periodic orbit (in black in Fig. 4b) is destroyed and only the global attractor of quiescence (equilibrium point) remains in the system (blue region in Fig. 2b, and black star in Fig. 4b). Then, during this pulse, all the orbits change their course to temporarily go towards the black star. Obviously, inactive neurons are the least affected by this pulse. Afterwards, the system evolves again with the black periodic orbit. While the inhibitory pulse is applied active neurons can be completely inhibited or just perturbed a bit. This effect depends on the length of the pulse and the position of the neuron in the nominal orbit. Later, during the excitatory pulse the black periodic orbit is replaced by the violet one (Fig. 4b), moving the parameters down in Fig. 2b. As we can see in Fig. 2b, this excitation increases the number of spikes and the length of the activity cycle. The neurons excited during the inactivity cycle immediately jumps into activity. This effect is clear in Fig. 4a, where both red and green neurons get activated by the excitatory pulse. In the case of the green neuron the combination of both pulses reduces dramatically its duty cycle. Neurons during the activity cycle get their number of spikes modified evolving like the violet orbit as long as the excitatory pulse is present. Finally, this pulse disappears leaving the system back to the nominal black attracting orbit. The perturbed CPG must evolve some time to reach the new synchronization pattern, but the spiking pattern of each neuron is quickly recovered. Obviously, these changes depend on the instant when the pulses are applied.

**Figure 5:**
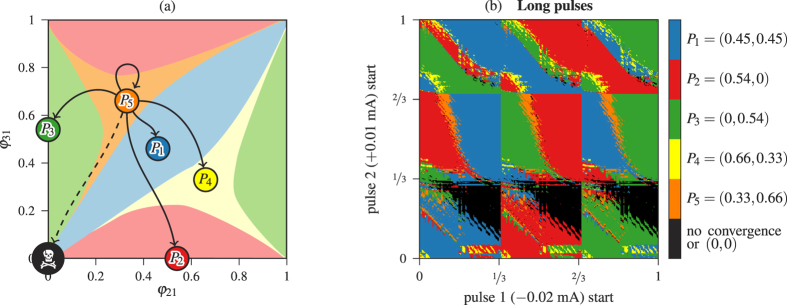
Control starting from the Orange fixed point *P*_5_ with pulses of length 9% of the period. (**a**) Possible changes of state. (**b**) Biparametric sweep of different instants of application of the two pulses showing the final stable states the network can reach.

Now we study in detail the control process. In Fig. 5a we represent the possible changes from the Orange stable fixed point (0.33, 0.66) to the rest of stable fixed points of the phase torus. The fixed point (0, 0) marked with a skull corresponds to the full synchronization of the three neurons. This pattern exists only because we are considering parameters to be equal among the three neurons. On the other hand, some authors hypothesize that synchronization is related to different pathologies such as Parkinson’s disease or epilepsy, leading to efforts to suppress synchronization on mathematical models of neuron networks^19^,^35^. In fact, they suggest that synchronization events obstruct information flow in a neuron network, and a external stimulus can re-establish desynchronization of the network^19^. We first consider equal neurons, but later we will allow small differences among them. From our starting pattern (Orange point), we intend to switch to any other pattern with the control technique.

Figure 5b shows a biparametric sweep of different instants of application of the two pulses control technique showing the final stable states the network can reach using the same color codes as Fig. 5a. The black color denotes non-convergence in the time of simulation or convergence to a point close to the fixed point (0, 0) (the skull point). Both pulses last 9% of the period length *P*. Considering time 0 when blue neuron starts bursting, and its period *P* as the unit, the abscissas represent the instant when the inhibitory pulse starts, and ordinates are the lapse between inhibitory and excitatory pulses. As we can see, there are combinations of pulses to jump to any possible stable fixed point. However, the yellow area in the plot is quite small and noisy, which makes difficult to aim to it. Note the 3-strip structure in both pulses due to the traveling-wave pattern of the 3-cell network of the starting point (Orange). This fact happens for all the simulations starting from a traveling-wave pattern.

**Figure 6:**
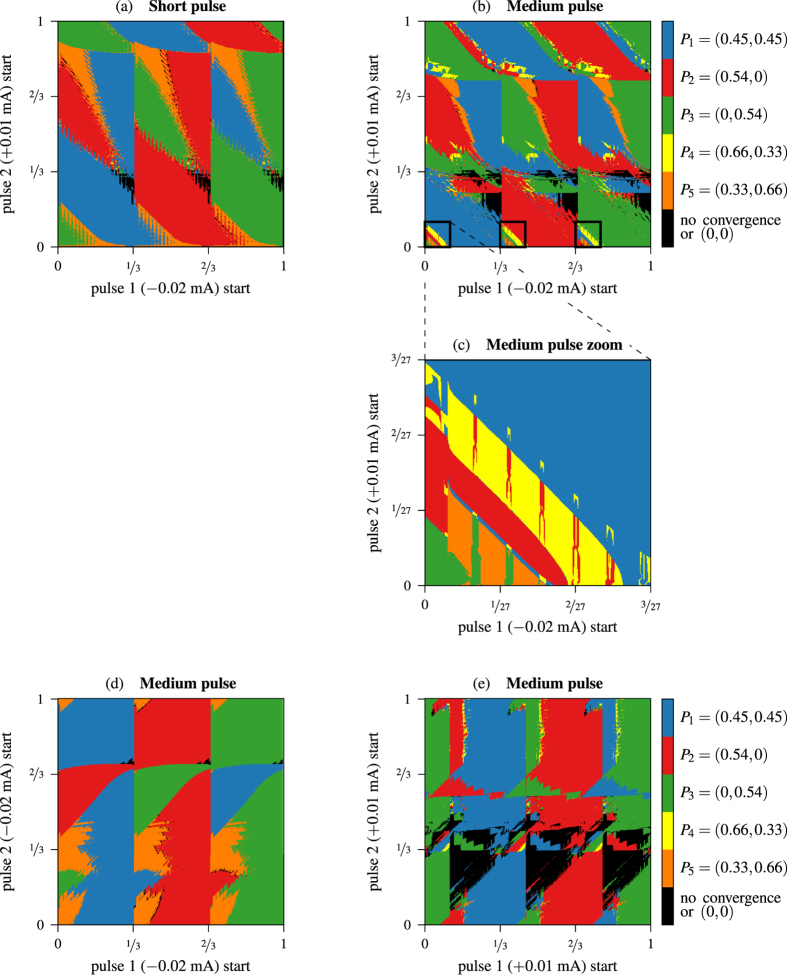
Biparametric sweep of different instants of application of the two pulses starting from the Orange fixed point *P*_5_ with: (**a**) inhibitory + excitatory pulses of length 1% (short pulse); (**b**,**c**) inhibitory + excitatory pulses of length 5% (medium pulse) of the period; (**d**) inhibitory + inhibitory pulses of length 5% (medium pulse); (**e**) and excitatory + excitatory pulses of length 5% (medium pulse).

In order to solve these problems we shorten the length of the pulses down to 1% of the period. Figure 6a shows the result of this strategy. It is clear that the plot is less noisy, but the yellow color has almost disappeared (just a couple of pixels). Besides, the non convergence zone is smaller compared with Fig. 5b, what means, of course, that larger pulses destabilizes more the systems creating larger transients in some regions or, in worst cases, full synchronous bursting. Finally, we adopt an “in between” strategy, using pulses of length 5% of the period. The result is presented in Fig. 6b. In this plot we show that the non convergent area is still small, and the yellow color appears in a less noisy area. Moreover, we have marked three small squares where all the colors coexist in a clearly defined area. There are other regions where it is possible to switch to most of the synchronization patterns, but the regions are more disordered and less sharp than the marked ones. In Fig. 6c we show a magnification of the leftmost square, where the five colors appear in sharp striplike patterns. Thus, it is possible to easily select a combination of pulse lengths that switches the network to the desired state. In other words, a suitable control strategy with reasonable short pulses allows to go to any synchronization pattern in this 3-cell CPG model. This is an interesting result since it opens new lines of global control of the network via short and weak currents in all the network avoiding unrealistic approaches (*ad hoc* currents for each neuron). To complete the analysis, we show in plot (d) the case of two inhibitory pulses and in plot (e) the case of two excitatory pulses of length 5% of the period. Note that case (d) is quite similar to case (a), whereas case (e) barely allows to go to a “traveling-wave” pattern (*P*_4,5_).

**Figure 7:**
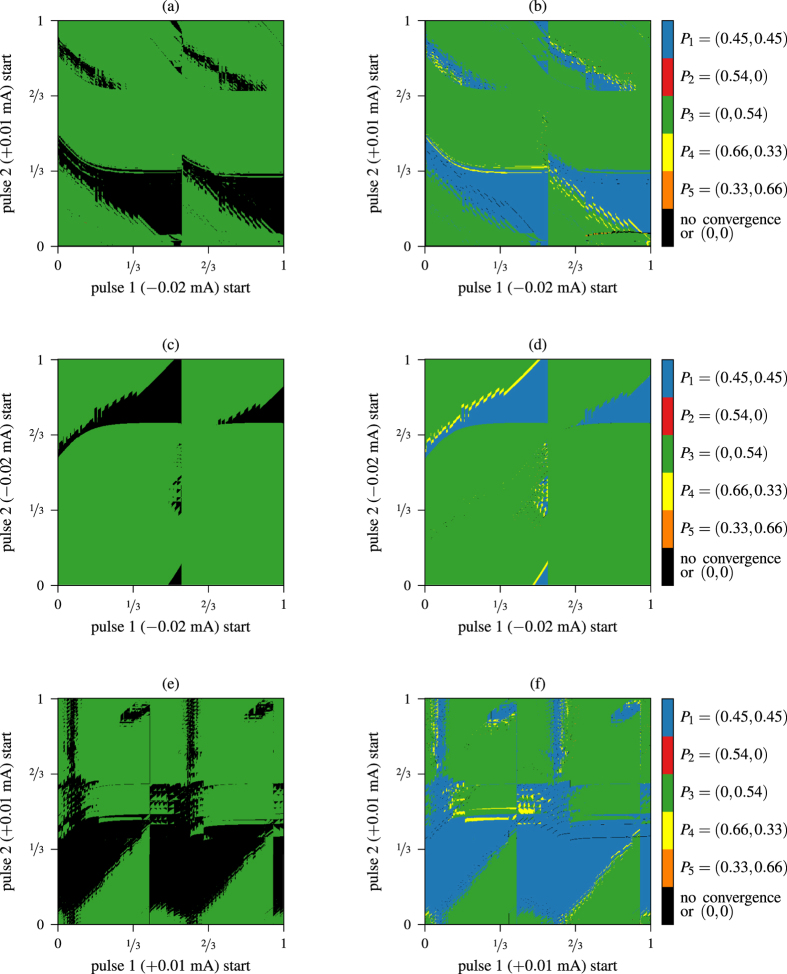
(Left column) Three equal neuron network and (right column) slightly modified neuron network simulations. Biparametric sweep of different instants of application of the two pulses of 9% of the period starting from the Green fixed point *P*_3_ with: (**a**,**b**) inhibitory + excitatory pulses; (**c**,**d**) inhibitory + inhibitory pulses; and (**e**,**f**) and excitatory + excitatory pulses.

Above, we have described the control strategies to jump from the Orange stable point *P*_5_ (see Fig. 5a). Due to the symmetry of the network, jumping from the Yellow point *P*_4_ is essentially the same as the already described situation. The other possible situation is to start from a pattern where two of the three neurons are synchronized, that is, to start from Blue *P*_1_, Red *P*_2_ or Green *P*_3_ points of Fig. 5a. Without loss of generality, let us describe the case of the Green point *P*_3_ shown in Fig. 7. Note the 2-strip structure due to the synchronicity pattern in two groups of the 3-cell network at the starting point (Green). Applying pulses lasting 9% of the period reveals that the only possible effects of this control strategy are either staying at the same point or falling into the full synchronization pattern (0, 0) (see left column plots of Fig. 7). But this is not a realistic situation since we are considering networks with perfectly equal neurons. Similar plots appear when shortening the length of the pulses.

In order to obtain useful control strategies, we take into account that real neurons may not have exactly equal intrinsic parameters as the ones used in the mathematical model (1). If we increase the parameter 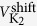 of the model by 1‱ of its value for the green neuron, and by 2‱ for the red one, we can observe that it is possible to jump from the Green fixed point to the Blue one (see right column of Fig. 7). Furthermore, there are tiny orange and yellow spots in the case of using opposite pulses, allowing us to return to the rich previous situation of the Orange state of Figs 5 and 6. So, allowing small differences among neurons (which is indeed a more realistic situation) we show how a small CPG can be effectively controlled to switch from any stable pattern to the desired one. The use of two inhibitory or excitatory pulses looks very similar to the case of opposite pulses, but the yellow area is slightly bigger, making aim to it easier. This is a preliminary work that opens control strategies for small CPGs, but more detailed analysis and theoretical studies are part of our open problems.

**Figure 8:**
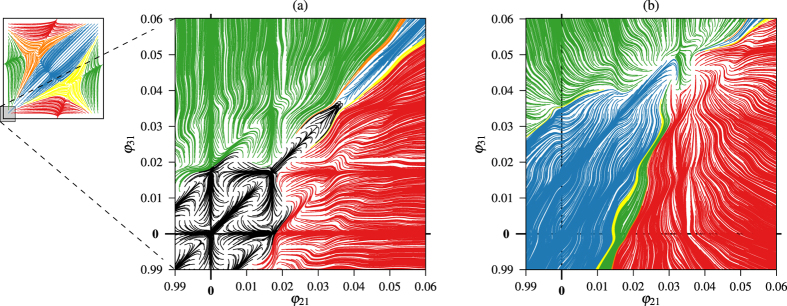
Neighbourhood of the complete synchronization state (0, 0) in the phase torus for (**a**) perfectly equal neurons and, (**b**) slightly perturbed neurons, increasing the parameter 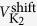 by 1‱ of its value for the green neuron, and by 2‱ for the red one. It is remarkable the absence of black basins.

Our remaining question is related to the origin of the change in behavior observed in Fig. 7 with small alterations in neurons. In Fig. 8 we plot two magnifications of the neighbourhood of the complete synchronization state (0, 0) (skull point) in the phase torus. Since the first pulse inhibits the neurons, lags between them decrease, moving the state of the network towards (0, 0) in the phase torus. In Fig. 8a we show the case of perfectly equal neurons. In this situation, close to the origin there are several equilibrium points in the black region (full synchrony pattern). Neither the control technique nor the evolution of the system itself can push the network towards another pattern. On the other hand, in Fig. 8b, where we show the case of slightly perturbed neurons, the black region has completely disappeared and there are “tracks” towards all the basins of attractions of the *P*_1−5_ fixed points. Moreover, the fact that the black basin containing the (0, 0) becomes now part of the blue basin, explains the very same phenomenon shown in Fig. 7. Therefore, the “realistic case” of slightly different neurons (or the presence of some “noise”) makes more robust the network allowing more options to control the CPG by means of two weak and short global pulses.

## Discussion

The discovery of the fact that several synchronization patterns of individual neurons are related to some serious neurological diseases (like Parkinson’s disease) was a remarkable advance and motivated numerous studies in techniques to avoid them. We show how, with a suitable combination of global inhibitory and/or excitatory stimuli of a neuron network, we can switch between different stable bursting patterns in small CPG neuron networks (in our case a 3-cell network). Other authors have used the approach of per-neuron stimulus, but since the distances within the CPG are tiny, this approach seems to be unrealistic. Our approach is based in global stimuli, a more realistic one, using two weak short pulses (inhibitory and/or excitatory), and so it avoids “dangerous” strong perturbations. We explain how a global stimulus modifies the intrinsic dynamics of each neuron (Fig. 4). We also examine how the instant where the pulses are applied at, determines the final state of the network (Figs 5, 6, 7). Remarkably, we exhibit *error-resistant pulse combinations* to switch between stable states, that is, small perturbations on when any of the pulses are applied does not alter the desired change. We also consider slightly different neurons in the same network obtaining more complete and accurate results, avoiding the full synchronization pattern. Moreover, we explain how these small differences, or noisy environments, explain the better performance of the control strategy.

It should be interesting for future research to explore other kind of stimuli and control techniques. For example, chemical modifications of some parameters, introducing smooth changes in the system instead of the discontinuities used in previous research. Besides, we expect that some of these ideas may be useful in larger networks, but in that case a previous development of new mathematical techniques to locate and represent the different synchronization patterns is required.

## Methods

The numerical integration of the differential equations of the mathematical model, to generate the data of each line of the phase torus, has been done using a embedded Runge-Kutta scheme of order 5 (dopri5(4) RK method) with dense output^36^ (to compute Poincaré sections) and variable-stepsize. Since the computation of each line is independent from any other, the global computation is completely parallelizable. We remark that we can take advantage of the latest computation devices such as multi-core CPUs, GPGPUs, Many Integrated Core coprocessors, etcetera. For computing the plots we have used a NVIDIA Tesla C2075 GPU-card, generating the data around 90 times faster than using a single core^37^.

The set of parameters used in the integration of the model (Eq. 1) is: 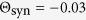, *E*_syn_ = −0.0625, *E*_Na_ = 0.045, 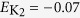, *E*_L_ = −0.046, *I*_app_ = 0.006, 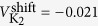, *g*_Na_ = 160.0, 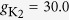, *g*_L_ = 8.0, *C* = 0.5, *τ*_Na_ = 0.0405, 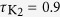. The value *g*_syn_ = 0.0007 is used for control results in the section that introduces the *control strategies via global stimuli*, and *g*_syn_ = 0.0004 for smooth visualization of phase space of the network in the section that describes the *Mathematical Neuronal Model*.

## Additional Information

**How to cite this article**: Lozano, Á. *et al.* Control strategies of 3-cell Central Pattern Generator via global stimuli. *Sci. Rep.* **6**, 23622; doi: 10.1038/srep23622 (2016).
